# Having lunch at the staff canteen and plant food consumption among 19–39-year-old Finnish employees

**DOI:** 10.1186/s12889-025-22445-5

**Published:** 2025-04-01

**Authors:** Elina Mauramo, Jatta Salmela, Noora Kanerva, Susanna Raulio, Tea Lallukka

**Affiliations:** 1 Department of Public Health, Faculty of Medicine, University of Helsinki, P.O. Box 20, Helsinki, 00014 Finland; 2 Department of Food and Nutrition, Faculty of Agriculture and Forestry, University of Helsinki, Helsinki, Finland; 3 Nutrition Unit, Department of Health, National Institute for Health and Welfare, Helsinki, Finland

**Keywords:** Employees, Workplace, Lunch, Plant foods, Socioeconomic

## Abstract

**Background:**

Having lunch at the staff canteen has been shown to be associated with daily food consumption and generally healthier food habits in employed populations but associations with the consumption of different types of plant foods have not been investigated. This study among Finnish municipal employees examined whether having lunch at the staff canteen is associated with the consumption of a range of plant foods including fruit, berries, fresh and cooked vegetables and wholegrain bread.

**Methods:**

Survey data from the Helsinki Health Study among female and male 19–39-year-old employees of the City of Helsinki, Finland, were used. The survey was conducted in 2017 (*N* = 5898, response rate 51.5%). A food frequency questionnaire was used to inquire about the overall consumption of food items during the last four weeks, with frequency categories ranging from ‘not during the past 4 weeks’ to ‘two times or more daily’. Variables of daily/non-daily consumption and consumption times/four weeks were formed for each plant food item. Having lunch at the staff canteen was used as a dichotomy of ‘yes/no’. Covariates included socioeconomic circumstances and working conditions. Logistic regression modelling was used for analysing the associations.

**Results:**

Employees who usually had lunch at the staff canteen (39%) consumed vegetables, but not berries, fruit or whole grain bread, more frequently than those who did not Having lunch at the staff canteen was associated with daily (vs. non-daily) consumption of both fresh vegetables (women OR 1.93, 95% CI 1.63–2.28; men OR 2.18, 1.68–2.83) and cooked vegetables (women OR 1.29, 1.11–1.51; men OR 1.75, 1.23–2.50). The observed associations remained after adjustments for socioeconomic circumstances and working conditions.

**Conclusions:**

Having lunch at the staff canteen was associated with more frequent fresh and cooked vegetable consumption. The results warrant further studies, including interventions, investigating whether and how staff canteens could promote the consumption of plant foods among employees.

**Supplementary Information:**

The online version contains supplementary material available at 10.1186/s12889-025-22445-5.

## Introduction

Consumption of plant foods, such as fresh vegetables, fruit, and wholegrain, is crucial for health promotion [1–3] and the prevention of obesity and chronic diseases [4–6]. However, the consumption level of plant foods does not reach international and national recommendations in high-income countries [7]. In Finland, according to the most recent national survey, only 14% of men and 22% of women reached the recommended daily minimum consumption level, 500 g, of vegetables, fruit and berries [8]. The consumption level of vegetables was similar across different age groups but for fruit and berries it was even lower among younger (18–44-year-old) than older (45–74-year-old) people.

Among the employed population, a large part of the daily waking hours is spent at work and workplace environments have thus been considered to have a large impact on the health behaviours, including dietary habits, of employees [9–12]. Food consumption during the workday has a major contribution to the overall daily food intake, as it has been estimated that among employees approximately a fourth or a third of the total daily energy intake comes from foods consumed during the workday, although there is likely to be cultural variation [8, 13, 14]. In Finland, lunch is often the main meal of the day and has been reported to contribute to daily food consumption in terms of energy intake more than dinner [8]. Thus, among employed people, the availability and accessibility of staff canteens, and the quality of the meals they offer, could influence daily food choices and the overall quality of the diet, including the use of plant foods [15, 16]. 

In Finland, staff canteen lunch meals offered by food service providers are required to be planned according to guidelines based on national dietary recommendations [17, 18]. Therefore, Finnish staff canteen lunches generally include fresh and cooked vegetables and wholegrain bread as staples, offered in a self-service buffet al.ong with hot main courses. There is some evidence from earlier Finnish survey studies suggesting that workplace canteens could contribute to overall healthier eating habits, such as more frequent fresh vegetable consumption, among the employed population [19, 20]. Also, workplace intervention studies from other countries have shown that staff canteens could have potential to promote a better quality of the diet, including a higher consumption level of fruit and vegetables [16, 21, 22]. 

In Finland, of the employed 25- to 64-year-old people, approximately a fifth of all men and a fourth of all women had lunch most often at a staff canteen according to the latest national survey [8]. The staff canteen lunch is subsidised and offered at a reduced fixed price, and the location is usually at the worksite or nearby. However, not all employees have access to a staff canteen. For those employees, who reported that they in general had access to a staff canteen, the figures for canteen utilisation were higher, with altogether 43% of women and 38% of men having lunch at the staff canteen [8]. Having lunch at the staff canteen was most common among the highest educated employees, but staff canteens nevertheless reached 20–30% of employees from lower educational groups as well. Staff canteens could thus be useful not only in the overall promotion of plant food use but also in terms of reducing the existing socioeconomic differences in the consumption of plant foods [23–26]. 

In this study among 19- to 39-year-old female and male municipal employees from Finland, our aim was to examine associations between having lunch at the staff canteen and the overall consumption frequency of various foods of plant origin. These included fruit, berries, fresh vegetables, cooked vegetables and wholegrain bread. In addition, we aimed to examine whether socioeconomic circumstances and working conditions would contribute to the associations between having lunch at the staff canteen and frequency of plant food consumption.

## Data and methods

### Survey data

**Fig. 1:**
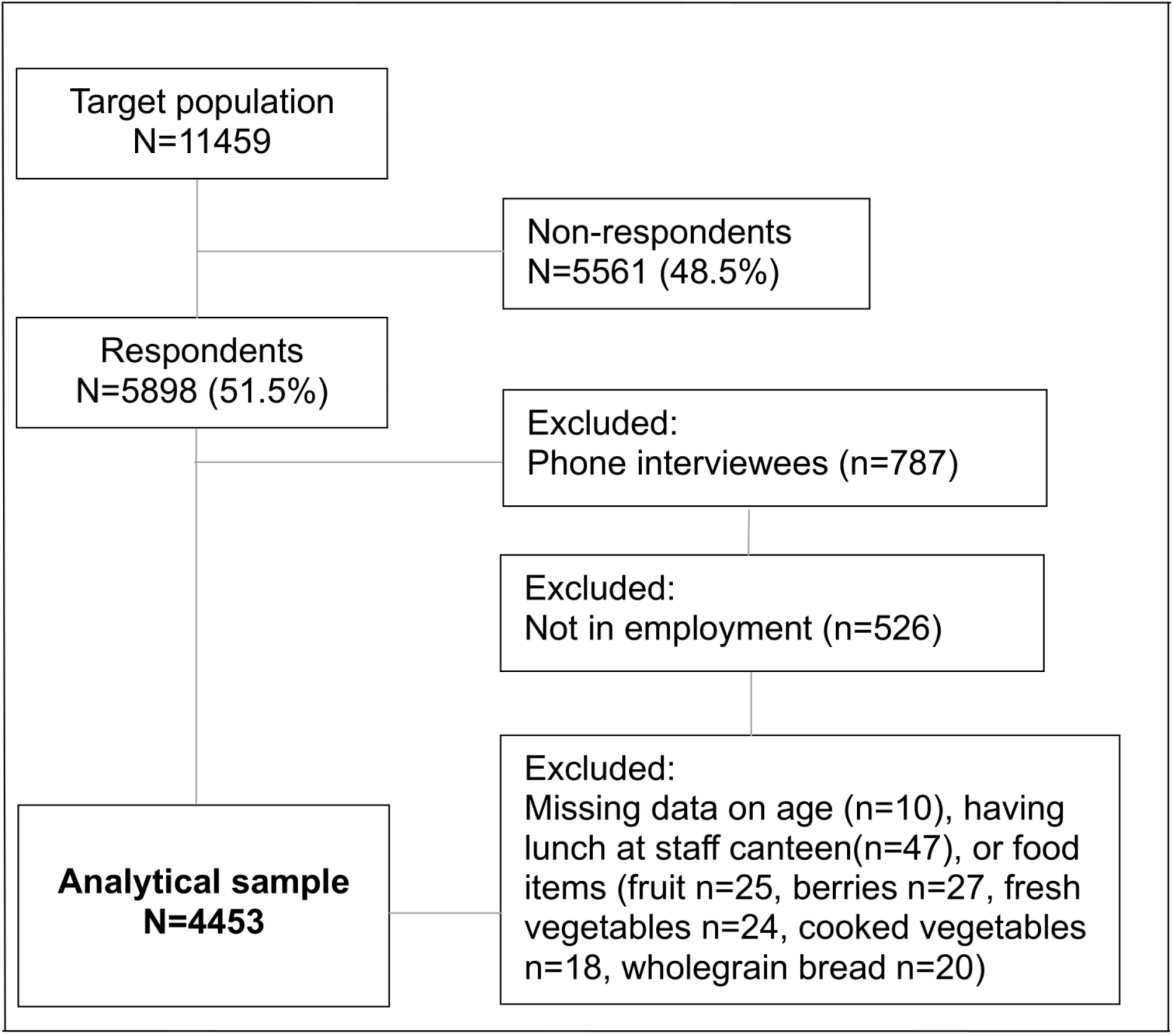
Flow chart of the selection of the analytical sample

We used survey data from the Helsinki Health Study (HHS) among 19- to 39-year-old employees [27]. The survey was conducted in 2017 among all those employees of the City of Helsinki, Finland, who were born in 1978 or later, and who at the time of the survey had been employed for 4 months or more with an employment contract of at least 50%.^27^ The survey was conducted using (1) online questionnaires, and (2) practically identical postal questionnaires among those who did not have an email address at work (however, it was possible to choose an online survey using a personal link provided with the mailed questionnaire), and (3) telephone interviews among those who did not respond to online or postal questionnaires. The overall response rate was 51.5% (*N* = 5898, 78.5% women). Since the telephone interviews were shortened and did not include all the measures used in this study, we excluded telephone interviewees. The HHS data collection has been described in more detail in a survey non-response analysis [27]. For the purposes of this study, we included participants who were employed at the time of the survey and had full information on having lunch at the staff canteen and the plant food items. The final number of participants included in the analyses was 4453. The flow chart of the sample selection is shown in Fig. 1. The ethical aspects of the Helsinki Health Study have been approved by the ethics committee at the Faculty of Medicine, University of Helsinki, and the City of Helsinki health authorities.

### Having lunch at the staff canteen

Having lunch at the staff canteen was assessed with the following question: [20] “Where do you usually have your lunch?” The response categories were: (1) at the staff canteen, (2) somewhere else at the workplace (e.g., in the coffee room), (3) in a restaurant (4) somewhere else than previously mentioned locations, and (5) I do not have lunch. Respondents who chose the first category were classified as having lunch at the staff canteen, and others as not having lunch at the staff canteen.

### Food frequency questionnaire

Consumption of different food items was measured with a 14-item food frequency questionnaire (FFQ). In this study, the utilised food items of the FFQ consisted of fruit, berries, fresh vegetables, cooked vegetables and wholegrain bread. Respondents were asked to estimate their consumption frequency of the different food items during the past four weeks, with the following frequency categories: ‘not during the past 4 weeks’, ‘1–3 times a month’, ‘once a week’, ‘2–4 times a week’, ‘5–6 times a week’, ‘once a day’ and ‘two times or more daily’. For each category, the average frequency per day was calculated and then multiplied by 28 days to produce the total number of consumption times per four weeks: 0, 2, 4, 12, 22, 28 and 56, following our previous studies [23, 28]. In addition, a dichotomous variable of daily versus non-daily consumption of each food item was formed.

### Covariates

Covariates included age, sex, socioeconomic circumstances [23] and working conditions. Education was divided into three levels: high (university degree), intermediate (matriculation or college examination) and basic (secondary or vocational school). The occupational class consisted of four hierarchical categories: managerial and professional (e.g., teachers and physicians), semi-professional (e.g., nurses and foremen), routine non-manual employees (e.g., childcare and elderly care workers) and manual workers (e.g., care assistants). The information was derived from the personnel register data for those who consented to register linkage (82%) and was completed from survey data. Household income was based on a question asking about the total typical monthly income of the respondent’s household. The monthly income was divided by household size and weighted according to the modified OECD equivalence scale which means that the respondent received a value of 1.0, other adults 0.5 and children 0.3 [29]. Four hierarchical income groups were formed with each of them consisting of approximately a quarter of the study population. Current economic difficulties were measured with two questions from Pearlin’s list of chronic strains: [30] ‘How much difficulty do you have in meeting the payment of bills?’ and ‘How often do you have enough money to buy the food or clothing you or your family need?’. Five response alternatives indicated the level of difficulties: ‘very little’ to ‘very great’ for the first question, and ‘always’ to ‘never’ for the second question. A combined variable was formed and categorised into no, occasional and frequent difficulties. Working conditions included (1) working time of 40 + hours per week versus less, (2) shift work versus normal working hours, (3) mental workload measured with a single question asking how heavy or light the respondent considered the work to be (very light / rather light / moderately heavy / very heavy), workplace atmosphere measured with a single question enquiring about the atmosphere and categorised into good and poor, and 5) workplace bullying measured with an instructed question about being bullied currently, previously or never.

### Statistical analyses

First, we calculated descriptive numbers and percentages. For the different plant food items, we computed the means of consumption frequency per four weeks among participants having or not having lunch at the staff canteen. Second, we fitted binary logistic regression models producing odds ratios (OR) with 95% confidence intervals (CI) to examine associations between having lunch at the staff canteen and daily versus non-daily consumption of the different food items, and the contribution of socioeconomic circumstances and working conditions. We first fitted age-adjusted base models (model 1). In the following models, we adjusted for socioeconomic circumstances and working conditions: first, education and occupational class (model 2), then household income and current economic difficulties (model 3), and, in the last models, working time and shift work (model 4) and mental workload, being bullied at the workplace and workplace atmosphere (model 5). All analyses were conducted separately for women and men. The analyses were performed using SAS statistical software version 9.4 (SAS Institute Inc., Cary, NC, USA).

## Results

The distribution of participants by having lunch at the staff canteen, plant food consumption and covariates is presented in Table 1. Of all participants, 39% reported that they usually had lunch at the staff canteen. Among the different plant foods, the highest prevalence of daily consumption, 70%, was found for fresh vegetables. For the other plant foods, prevalence percentages of daily consumption were 46% for fruit, 39% for wholegrain bread, and 25% for berries and cooked vegetables.

For each food item, daily and non-daily consumption and mean consumption frequency, that is consumption times per four weeks, are shown in Table 2, by having/not having lunch at the staff canteen. Among both women and men, participants who reported having lunch at the staff canteen had a clearly higher prevalence of daily consumption as well as higher mean consumption frequency of fresh vegetables and cooked vegetables than participants who did not have lunch at the staff canteen. For fresh vegetables, the mean consumption times per four weeks were 39 in women and 36 in men having lunch at the staff canteen vs. 36 in women and 26 in men not having lunch at the staff canteen (*p* < 0.001 for both sexes). For cooked vegetables, the respective numbers were 19 vs. 16 in women (*p* < 0.001) and 16 vs. 12 in men (*p* < 0.001).

**Table 1:** Distribution (*N*, %) of participants by having lunch at the staff canteen, plant food consumption and covariates

		*N*	%
**Having lunch at the staff canteen**	Yes	1737	39
	No	2716	61
**Plant food consumption**			
Fruit	Daily	2053	46
	Non-daily	2400	54
Berries	Daily	1100	25
	Non-daily	3353	75
Fresh vegetables	Daily	3097	70
	Non-daily	1356	30
Cooked vegetables	Daily	1109	25
	Non-daily	3344	75
Wholegrain bread	Daily	1734	39
	Non-daily	2719	61
**Sex**	Women	3486	78
	Men	967	22
**Age**	19–29	1443	32
	30–40	3010	68
**Education**	High	1328	30
	Intermediate	1603	36
	Low	1571	34
**Occupational class**	Professional	1249	28
	Semi-professional	1739	39
	Routine non-manual	1198	27
	Manual worker	244	6
**Household income**	Highest quartile	894	20
	2nd highest quartile	1141	26
	3rd highest quartile	1037	23
	Lowest quartile	1356	31
**Current economic difficulties**	No	2031	46
	Occasional	2024	45
	Frequent	387	9
**Shift work**	No	3117	72
	Yes	1236	28
**Working time**	Less than 40 h/week	3586	81
	40 h or more /week	867	19
**Mental workload**	Low	3642	82
	High	772	18
**Being bullied at workplace**	No	2897	66
	Yes, in previous workplace	907	20
	Yes, in current workplace	599	14
**Workplace atmosphere**	Good	3229	73
	Less than good	1193	27
**All**		4453	100

**Table 2:** Plant food daily/non-daily and mean consumption, by having/not having lunch at the staff canteen, women and men

		Women			Men	
	Having lunch at the staff canteen	Not having lunch at the staff canteen	*P*-values	Having lunch at the staff canteen	Not having lunch at the staff canteen	*P*-values
**Fruit**	N, %			N, %		
Daily	649 (50.3)	1112 (50.6)	0.85	130 (29.1)	162 (31.1)	0.48
Non-daily	641 (49.7)	1084 (49.4)		317 (70.9)	358 (68.9)	
	Mean, SD^a^			Mean, SD^a^		
Times/4 weeks	26.7 (17.3)	26.9 (18.3)	0.684	18.8 (16.1)	18.2 (16.1)	0.622
**Berries**	N, %			N, %		
Daily	352 (27.3)	626 (28.5)	0.44	53 (11.9)	69 (13.3)	0.51
Non-daily	938 (72.7)	1570 (71.5)		394 (88.1)	451 (86.7)	
	Mean, SD^a^			Mean, SD^a^		
Times/4 weeks	15.9 (14.0)	16.7 (15.6)	0.131	10.7 (10.7)	10.2 (11.9)	0.498
**Fresh vegetables**	N, %			N, %		
Daily	1056 (81.9)	1530 (69.7)	< 0.001	283 (63.3)	228 (43.9)	< 0.001
Non-daily	234 (18.1)	666 (30.3)		164 (36.7)	292 (56.2)	
	Mean, SD^a^			Mean, SD^a^		
Times/4 weeks	39.1 (16.5)	36.1 (18.3)	< 0.001	30.6 (15.8)	26.0 (17.8)	< 0.001
**Cooked vegetables**	N, %			N, %		
Daily	401 (31.1)	559 (25.5)	< 0.001	87 (19.5)	62 (11.9)	0.001
Non-daily	889 (68.9)	1637 (74.5)		360 (80.5)	458 (88.1)	
	Mean, SD^a^			Mean, SD^a^		
Times/4 weeks	18.6 (14.5)	16.8 (15.5)	< 0.001	15.5 (12.5)	11.5 (11.9)	< 0.001
**Wholegrain bread**	N, %			N, %		
Daily	532 (41.2)	867 (39.5)	0.306	156 (34.9)	179 (34.4)	0.877
Non-daily	758 (58.8)	1329 (60.5)		291 (65.1)	341 (65.6)	
	Mean, SD^a^			Mean, SD^a^		
Times/4 weeks	22.3 (17.1)	21.3 (17.9)	0.088	20.7 (16.6)	21.2 (18.7)	0.650

^a^ SD = standard deviation

In the logistic regression analyses, having lunch at the staff canteen was associated with the consumption of fresh and cooked vegetables among both women (Table 3) and men (Table 4). Among women, those who usually had lunch at the staff canteen, compared to those who did not, were almost twice as likely to consume fresh vegetables daily (OR 1.93, 95% CI 1.63–2.28). A weaker association was also observed for cooked vegetables (OR 1.29, CI 1.11–1.51). Among men, similar but somewhat stronger associations were found for fresh (OR 2.18, CI 1.68–2.83) and cooked (OR 1.75, CI 1.23–2.50) vegetables. In model 2, among both women (Table 3) and men (Table 4), adjustments for education and occupational class attenuated the associations observed for fresh and cooked vegetables to some degree but they remained statistically significant. For fresh vegetables, slight attenuation was also caused by adjustment for household income and economic difficulties in model 3 and for working time and shift work in model 4, among both women and men. For the other food items, that is fruit, berries and wholegrain bread, no associations were observed.

**Table 3:** Having lunch at the staff canteen and plant food daily consumption, women. Odds ratios (OR) with 95% confidence intervals (CI)

	Model 1 (M1):	MODEL 2:	MODEL 3:	MODEL 4:	MODEL 5:
	Age-adjusted	M1 + education,	M1 + household income,	M1 + working time,	M1 + mental workload, being
		occupational class	economic difficulties	shift work	bullied at workplace, workplace atmosphere
	OR (95% CI)	OR (95% CI)	OR (95% CI)	OR (95% CI)	OR (95% CI)
**Fruit**					
Having lunch at the staff canteen					
No (ref.)	1.00	1.00	1.00	1.00	1.00
Yes	0.98 (0.85–1.12)	0.87 (0.75–1.01)	0.94 (0.82–1.08)	0.94 (0.81–1.08)	0.98 (0.85–1.12)
**Berries**					
Having lunch at the staff canteen					
No (ref.)	1.00	1.00	1.00	1.00	1.00
Yes	0.94 (0.80–1.09)	0.86 (0.73–1.02)	0.89 (0.76–1.04)	0.91 (0.78–1.07)	0.92 (0.79–1.08)
**Fresh vegetables**					
Having lunch at the staff canteen					
No (ref.)	1.00	1.00	1.00	1.00	1.00
Yes	1.93 (1.63–2.28)	1.76 (1.48–2.10)	1.83 (1.54–2.17)	1.73 (1.45–2.06)	1.90 (1.60–2.26)
**Cooked vegetables**					
Having lunch at the staff canteen					
No (ref.)	1.00	1.00	1.00	1.00	1.00
Yes	1.29 (1.11–1.51)	1.20 (1.03–1.41)	1.26 (1.08–1.47)	1.33 (1.14–1.56)	1.28 (1.10–1.50)
**Wholegrain bread**					
Having lunch at the staff canteen					
No (ref.)	1.00	1.00	1.00	1.00	1.00
Yes	1.05 (0.91–1.21)	1.07 (0.93–1.24)	1.04 (0.91–1.20)	1.06 (0.92–1.23)	1.05 (0.91–1.21)

**Table 4:** Having lunch at the staff canteen and plant food daily consumption, men. Odds ratios with 95% confidence intervals

	Model 1 (M1):	MODEL 2:	MODEL 3:	MODEL 4:	MODEL 5:
	Age-adjusted	M1 + education,	M1 + household income,	M1 + working time,	M1 + mental workload, being
		occupational class	economic difficulties	shift work	bullied at workplace, workplace atmosphere
	OR (95% CI)	OR (95% CI)	OR (95% CI)	OR (95% CI)	OR (95% CI)
**Fruit**					
Having lunch at the staff canteen					
No (ref.)	1.00	1.00	1.00	1.00	1.00
Yes	0.89 (0.68–1.78)	0.77 (0.57–1.04)	0.86 (0.65–1.14)	0.89 (0.67–1.20)	0.92 (0.69–1.21)
**Berries**					
Having lunch at the staff canteen					
No (ref.)	1.00	1.00	1.00	1.00	1.00
Yes	0.88 (0.60–1.29)	0.89 (0.59–1.34)	0.87 (0.59–1.27)	0.96 (0.64–1.43)	0.90 (0.61–1.33)
**Fresh vegetables**					
Having lunch at the staff canteen					
No (ref.)	1.00	1.00	1.00	1.00	1.00
Yes	2.18 (1.68–2.83)	1.78 (1.35–2.36)	2.08 (1.59–2.71)	1.93 (1.47–2.53)	2.22 (1.70–2.89)
**Cooked vegetables**					
Having lunch at the staff canteen					
No (ref.)	1.00	1.00	1.00	1.00	1.00
Yes	1.75 (1.23–2.50)	1.50 (1.03–2.20)	1.69 (1.18–2.42)	1.74 (1.20–2.54)	1.82 (1.27–2.62)
**Wholegrain bread**					
Having lunch at the staff canteen					
No (ref.)	1.00	1.00	1.00	1.00	1.00
Yes	1.00 (0.77–1.31)	1.06 (0.80–1.41)	0.97 (0.74–1.27)	1.03 (0.77–1.36)	1.00 (0.76–1.31)

## Discussion

This study examined associations between having lunch at the staff canteen and consumption of a range of plant foods among 19- to 39-year-old employees of the City of Helsinki, Finland. The main result of the study was that having lunch at the staff canteen was associated with more frequent consumption of fresh and cooked vegetables among both women and men. The strongest associations were found for fresh vegetables. For the other examined plant foods, that is fruit, berries, and wholegrain bread, no associations were observed. The associations were slightly affected by adjustments for socioeconomic circumstances and working conditions, but they remained clear.

First, the observed associations between having lunch at the staff canteen and fresh and cooked vegetable consumption were in general supportive of earlier observations among Finnish employees. Previous Finnish studies have suggested having lunch at the staff canteen to be associated with a diet pattern that is overall healthier and more in line with national recommendations, including a higher level of vegetable consumption, compared to having lunch elsewhere [19, 20]. This study produced novel information on the subject by focusing on comparing a range of plant foods. The results showed that especially fresh and cooked vegetable consumption, in comparison to fruit, berry and wholegrain bread consumption, which showed negligible associations, is more frequent among employees who usually have lunch at the staff canteen. However, it should be noted, that whether the observed higher consumption frequency originates from consuming the food at the lunchtime or not, cannot be confirmed from our results as we examined the overall daily plant food consumption. Lunchtime consumption is at least partly probable, as Finnish staff canteen lunches are to a large part planned according to the national dietary recommendations to ensure nutritional quality and generally include fresh and cooked vegetables, although offering vegetables does not always ensure they are consumed [17, 18]. Staff canteen lunches in Finland also generally include wholegrain bread, but fruit and berries less often. A Danish study comparing meal quality at 15 staff canteens found an estimated mean intake to be 133 g of vegetables and fruit per meal [15]. Such concrete measurements and observations would be warranted to confirm the consumption of different types of food as well as consumed amounts at the workplace lunch in detail.

Second, the documented associations between having lunch at the staff canteen and more frequent fresh and cooked vegetable consumption were parallel among women and men, but even somewhat stronger among men. In a previous study among ageing municipal employees, a lower long-term level of fruit and vegetable consumption frequency was observed among men compared to women [28]. A question could be raised on a possible difference between women and men in the importance of having lunch at the staff canteen as a contributor to daily food intake. Could the staff canteen be even more important to men’s overall daily vegetable consumption? It has been shown that women are in general more often health conscious and more likely to make healthy food choices compared to men [31], and it could thus be reasoned that in terms of increased daily consumption, men might possibly profit more from the staff canteen meals offering plant foods. In addition, differences in the occupations between women and men could, especially among the manual workers, affect the availability and accessibility of the staff canteen in the first place, as well as the social norms related to its use. Male participants in our study worked more often, for example, in construction and public transportation whereas female participants worked in the social and healthcare sector. Furthermore, it should be noted, that the number of men was small in our study, and confidence intervals were wider (Table 4) for men than women. Thus, if the number of men had been higher, the results might have shown even stronger associations for men.

Third, adjusting for education and occupational class had attenuating effects on the associations which, however, remained. It is known from previous studies that both having lunch at the staff canteen and the consumption of plant foods differ between socioeconomic groups [8, 20, 23]. Thus, it could have been expected that socioeconomic circumstances would have had a greater contributing role. Nevertheless, with regard to the socioeconomic differences, it should be noted that those employees who report that they usually have lunch at the staff canteen are already a selected population. In this study, we did not have information on whether the participant would have access to a staff canteen, but it is known that the availability and accessibility are in general better among the higher socioeconomic groups. Barriers to eating at the staff canteen, such as price, location, or social factors, affect employees with lower education, in lower occupational classes and income groups more than the socioeconomically advantaged employees [32]. Also, unfavourable work characteristics such has shift work could affect the access to and availability of a staff canteen lunch among employees in lower socioeconomic groups more than those in higher positions.

Fourth, the contribution of working conditions was also examined but only weak and inconsistent effects on the associations were found. Only the adjustment for working time and shift work very slightly attenuated the association observed for fresh vegetable consumption. Having lunch at the staff canteen could vary according to working conditions, which is shown in an additional table (see Additional File 1). An earlier Finnish study found regular day work as well as low mental strain at work to be associated with the use of the staff canteen [33]. These kinds of work-related factors could also be associated with food consumption habits in general, and thus could have been expected to have a somewhat greater contribution to the results of the current study.

Overall, the results suggest that further studies are warranted concerning the meal quality of staff canteens and their exact contribution to employees’ plant food consumption. Future studies should also consider other employee groups including municipal employees from different age groups as well as employees elsewhere in the public sector and in the private sector. Specifically, employees in manual work would be a group in which there could be the largest potential in terms of increasing both the access to staff canteens and the overall consumption level of plant foods to promote employee health and to reduce socioeconomic differences in health [23, 25, 26]. In addition, investigations, including intervention studies, are needed on the concrete ways in which plant foods could be promoted in workplaces and among employees most efficiently. Qualitative methods could provide means to investigate factors that employees themselves consider as affecting their use of the staff canteen and their food choices. Different kinds of interventions have already been made internationally which could be applied in the working places for increasing plant food use as a part of a health promoting diet [15, 16, 22, 34, 35]. Results from such intervention studies need to be put into practice to suit different kinds of employee groups and working environments.

### Methodological considerations

This cross-sectional study was based on survey data among young and midlife municipal employees. The study population can be considered as representative of the target population, the female and male 19- to 39-year-old employees of the City of Helsinki, Finland. The proportion of female employees was high among the respondents, 78.5%, corresponding to the overall sex distribution among the employees of the City of Helsinki. According to a non-response analysis, differences between the target and study populations have been documented to be small in the distribution of socioeconomic and health factors [27]. In general, the results are likely to be, with caution, generalisable to young and midlife employees in the municipal sector in Finland, and possibly also elsewhere in the public sector. With regard to the measure of food consumption, a 14-item food frequency questionnaire, short FFQs have in previous studies been shown to be suitable as measures of frequently consumed foods [36]. 

The limitations include the characteristics of the survey data and the study population. The majority of the participants were women, similarly to the population employed in the Finnish municipal sector in general. This causes restrictions to the statistical power among men and should be taken into account when making generalisations. The survey measures used also cause limitations: the FFQ was not validated and it did not include portion sizes. Therefore, it was not possible to assess food consumption quantities in this study. It has been shown, however, that the contribution of portion size questions in FFQs to the food intake variance may be negligible [37]. Furthermore, the FFQ did not include detailed information on different types of fruit and vegetables, for example, but consisted of broader food group categories. However, to get the most information out of these questions, we examined the available food items separately, including “fruit” and “berries”, which are often summed together in studies. With regard to the information on having lunch at the staff canteen, the survey did not include questions concerning the access to or availability of a staff canteen. Therefore, we were not able to take into account whether or not those participants, who did not usually have lunch at a staff canteen, would have had access to a staff canteen. Also, when interpreting the results, it should be taken into account, that the wording of the question on usually having lunch at a staff canteen or elsewhere leaves room for some uncertainty as no detailed frequency information was available. In addition, the general cautions which are warranted when interpreting results that are based on self-reported data, due to, for example, under or over-reporting, apply to this study. Especially, over-reporting is a possibility with regard to the consumption of plant foods. Also, it should be noted that causality cannot be determined with cross-sectional survey data.

### Conclusions

This study among Finnish municipal employees found having lunch at the staff canteen to be associated with more frequent consumption of fresh and cooked vegetables but not with the consumption of fruit, berries and wholegrain bread. Staff canteens could provide opportunities to increase the consumption of plant foods among the employed population, and the results of this study warrant interventions that investigate how to apply such results in practice.

## Electronic supplementary material

Below is the link to the electronic supplementary material.

Supplementary Material 1

## Data Availability

No datasets were generated or analysed during the current study.

## Abbreviations

HHS: Helsinki health study
FFQ: Food frequency questionnaire
OR: Odds ratio
CI: Confidence interval
