# Supplementary material for: Having lunch at the staff canteen and plant food consumption among 19–39-year-old Finnish employees
Source: BMC Public Health. 2025 Apr 1;25:1237. doi: 10.1186/s12889-025-22445-5 (PMC11963650; doi:10.1186/s12889-025-22445-5)
Supplement: Supplementary file 1 — Supplementary Material 1 [file 12889_2025_22445_MOESM1_ESM.docx]

Appendix Table 1. Having or not having lunch at staff canteen (n, %) by psychosocial working conditions.

|  | **Having lunch at staff canteen** | **Not having lunch at staff canteen** | **P-values** |
| --- | --- | --- | --- |
|  |  |  |  |
|  | N, % | N, % |  |
| **Shift work** |  |  |  |
| No | 447 (46.1) | 1681 (53.9) | <0.001 |
| Yes | 270 (21.8) | 966 (78.2) |  |
| **Working time** |  |  |  |
| Less than 40 hours/week | 1357 (37.8) | 2229 (62.2) | 0.001 |
| 40 hours or more /week | 380 (43.8) | 487 (56.2) |  |
| **Mental workload** |  |  |  |
| Low | 1413 (38.8) | 308 (39.9) | 0.570 |
| High | 2229 (61.2) | 464 (60.1) |  |
| **Being bullied at workplace** |  |  |  |
| No | 1202 (41.5) | 1695 (48.4) | <0.001 |
| Yes, in previous workplace | 312 (34.4) | 595 (65.6) |  |
| Yes, in current workplace | 206 (34.4) | 393 (65.6) |  |
| **Workplace atmosphere** |  |  |  |
| Good | 1267 (39.2) | 1962 (60.8) | 0.440 |
| Less than good | 453 (38.0) | 740 (62.0) |  |
|  |  |  |  |
